# Programming effects of maternal and gestational obesity on offspring metabolism and metabolic inflammation

**DOI:** 10.1038/s41598-019-52583-x

**Published:** 2019-11-05

**Authors:** E. Chang, H. Hafner, M. Varghese, C. Griffin, J. Clemente, M. Islam, Z. Carlson, A. Zhu, L. Hak, S. Abrishami, B. Gregg, K. Singer

**Affiliations:** Department of Pediatrics, Division of Diabetes, Endocrinology, and Metabolism, University of Michigan Medical School, Ann Arbor, MI USA

**Keywords:** Developmental biology, Obesity

## Abstract

With the increasing prevalence of obesity in women of reproductive age there is a need to understand the ramifications of this on offspring. The purpose of this study is to investigate the programming effects of maternal obesity during preconception and the preconception/gestational period on adiposity and adipose tissue inflammation in offspring using an animal model. Adult female C57Bl/6J mice were assigned either normal diet, high fat diet (HFD) prior to pregnancy, or HFD prior to and through pregnancy. Some offspring were maintained on normal diet while others started HFD later in life. Offspring were assessed for body composition and metabolic responses. Lipid storing tissues were evaluated for expansion and inflammation. Male offspring from the preconception group had the greatest weight gain, most subcutaneous adipose tissue, and largest liver mass when introduced to postnatal HFD. Male offspring of the preconception/gestation group had worsened glucose tolerance and an increase in resident (CD11c^−^) adipose tissue macrophages (ATMs) when exposed to postnatal HFD. Female offspring had no significant difference in any parameter between the diet treatment groups. In conclusion, this study demonstrates that prenatal and pregnancy windows have independent programming effects on offspring. Preconception exposure affects body composition and adiposity while gestation exposure affects metabolism and tissue immune cell phenotypes.

## Introduction

The prevalence of obesity has increased in the United States for all populations including women of reproductive age. Obesity during pregnancy is concerning due to an increased risk for pregnancy complications, such as gestational diabetes and preeclampsia, as well as delivery and postnatal complications^1,2^. However, the prevalence of childhood obesity is also rising on a global scale^3^. Childhood obesity can be framed as a condition in which growth, development, the environment, and genetic predisposition are intertwined^4^. The traditional dogma has assumed obesity occurred because of an imbalance between the caloric intake versus the caloric output^5^. However, it is now evident that there are individuals who are prone to developing obesity and its associated metabolic complications due to an underlying vulnerability^6^. There is mounting evidence that developmental programming plays a role in regulating this vulnerability in offspring^7^. Maternal obesity not only causes short-term complications but also has long-term effects on offspring body composition, metabolism, and predisposition to obesity, insulin resistance, and dyslipidemia^8^.

There is a growing focus on the specific effects of maternal high fat diet (HFD) induced obesity on offspring in both animal and human studies. A large body of studies suggest that there are critical windows of development (preconception, early gestation, late gestation) in which maternal obesity can induce programming effects on offspring physiology and organ development^9,10^. Fetuses removed from obese mice as early as gestational day 18 showed increased subcutaneous fat with more adipocyte hypertrophy and decreased GLUT-4 gene expression suggesting a decrease in fetal insulin sensitivity, when compared to fetuses obtained from lean C57BL/6J mice^11^. Using a large animal sheep model with overnutrition exposure from 10 weeks prior to conception up to mid-gestation, fetuses collected at mid-gestation from the overweight/obese mothers had greater adiposity compared to the normal control^12^. When comparing the fetuses of overweight mothers to obese mothers, the fetuses from the obese mothers had a higher body weight, pancreas weight, and liver weight^12^.

However, further studies are showing that the other windows may also be critically important to offspring health. One study using female C57BL/6J mice maintained on an obesogenic diet for 6 weeks before mating and through lactation had offspring with increased food intake, adiposity, hypertension, and insulin resistance compared to control mice born from female mice not on an obesogenic diet^13^. Offspring born from C57BL/6J female mice exposed to HFD during gestation only and that remained on standard diet ended up with greater body weight, hyperglycemia, hypertension, fatty liver, and insulin resistance as adults, when compared to offspring born from mice with standard diet exposure^14,15^. In humans, studies examining siblings who were born before and after their mother had bariatric surgery to reduce adiposity showed that siblings born post-surgery are leaner, more insulin sensitive, and have lower blood pressure compared to the sibling born before bariatric surgery^16,17^.

**Figure 1:**
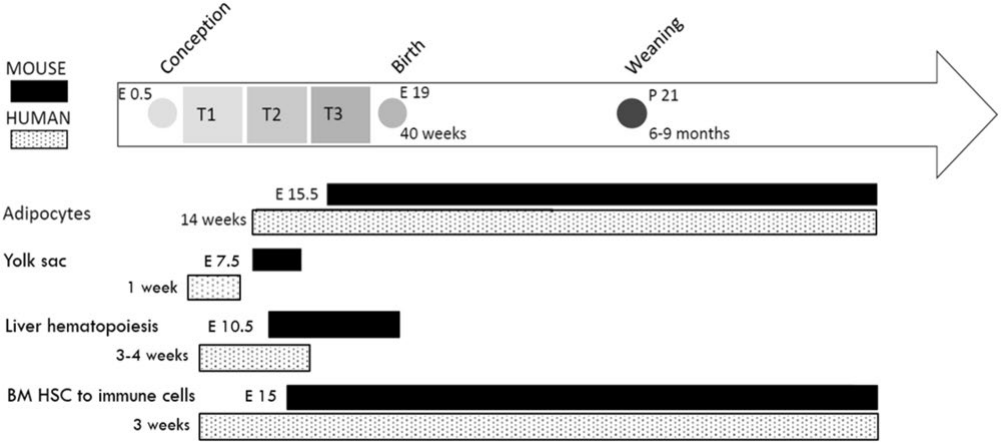
Developmental timeline of human and mouse tissues and cells. This shows the developmental timeline for various tissues and cells from conception through the lactation period. Cells and tissues include: adipocytes, yolk sac (site of early formation of blood), liver hematopoiesis (main site of hematopoietic stem cell expansion and differentiation), and bone marrow hematopoietic stem cell (BM HSC) differentiating into immune cells (e.g. macrophages). This figure was adapted from another of our studies^9^.

These studies demonstrate that there are critical windows of programming which are likely related to the stages of organogenesis throughout the gestation period (Fig. 1)^9^. Different timing of exposure to an obesogenic environment could lead to altered phenotypes in the offspring depending on what cells and tissues are developing at that time. Adipocytes and immune cells begin to form during mid to late pregnancy in both mice and humans^18^. These are two important cell types for global metabolism because of their involvement with nutrient storage, insulin resistance and meta-inflammation^19^. Adipose tissue is a reservoir for adipocytes, pre-adipocytes, fibroblasts, endothelial cells and immune cells in the stromal vascular fraction (SVF) and plays a significant role in global metabolism^20^. It stores and releases lipids, takes up glucose in response to insulin, and produces adipokines^21,22^. Adipose tissue therefore serves as a nexus between inflammation and obesity-related dysfunction and cardiovascular disease^16^.

Adipose tissue dysfunction has several physiologic effects. For example high levels of free fatty acid released from visceral adipose tissue during HFD exposure induces an acute host inflammatory response that is meant to restore metabolic homeostasis^23^. However, if the inflammatory response becomes chronic due to an ongoing obesogenic environment, there is disruption to the homeostatic system that regulates physiologic variables, such as blood glucose, and an imbalance of adipose tissue-derived cytokines and adipokines, promoting a further proinflammatory state^4,24–26^. A hallmark of adipose tissue inflammation is infiltration of immune cells such as monocytes and macrophages in response to expansion, adipocyte death, and chemokine signals^27^. This raises the question of whether maternal obesity causes programming changes that influence metabolic inflammation in developing adipose tissue and thereby alters the offspring phenotype.

This study was designed to determine the effects of HFD exposure to the offspring of C57BL/6J female mice during specific developmental windows, prior to conception (Pre) or from preconception through gestation (P/G). Developmental programming paradigms often require a “second-hit” which involves the introduction of a stressor after the initial programming event to determine if latent programmed phenotypes emerge under situations of metabolic stress. A HFD trigger is a clinically relevant stressor for subjects who have encountered developmental programming stressors during development^28^. The study’s hypothesis is that maternal HFD during the preconception period and the preconception/gestational period programs offspring adiposity, metabolism, and adipose tissue inflammation and that these programmed changes are exacerbated by a “second hit” of HFD later in life.

## Materials and Methods

### Experimental animals

**Figure 2:**
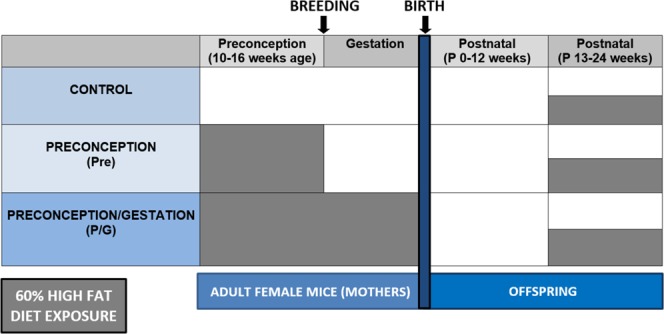
Experimental diet groups and HFD exposure timeline. Control group were only on normal diet. The Pre group (Preconception) had mothers with HFD exposure only prior to pregnancy. The P/G (Preconception/Gestation) group had mothers with HFD exposure 6 weeks prior to and through pregnancy. All offspring were on ND for the first 12 weeks of life. At 13 weeks of age, half of the offspring from each diet group were maintained on a normal diet while the other half were switched to HFD.

Virgin female and male wild type C57BL/6J mice were purchased from Jackson Laboratories at 5 weeks of age. The mice habituated and were fed *ad libitum* a control normal diet (ND) consisting of 13% calories from fat (5001; Laboratory Diet, St. Louis, MO). Animals were housed in a specific pathogen-free facility with a 12-hour light/12-hour dark cycle and were allowed free access to food and water. Animal protocols followed the Institute of Laboratory Animal Research *Guide for the Care and Use of Laboratory Animals* and approved by the University Committee on Use and Care of Animals at the University of Michigan. At 10 weeks old, the Pre and P/G females were randomized to HFD consisting of 60% of calories from fat (D12492, Research Diets, New Brunswick, NJ). Figure 2 shows the different windows of HFD exposure. All the remaining females remained on control ND and were designated as the control group. All males remained on control ND. After 6 weeks on their respective diets, breeding cages were established where one female was paired with one male. Male mice in the P/G group were placed in the same cage as females for mating and hence exposed to the same HFD as the females in that group. However, their exposure was only during the short window required to detect a copulatory plug (less than 5 days), hence it is not likely that spermatozoa were affected since the maturation of male sperm is approximately 35 days^29^.

The first day of pregnancy was determined by the presence of a vaginal plug in the morning. At this point, the Pre mice were switched to ND. The P/G mice continued HFD through gestation. During pregnancy, maternal weight was recorded weekly. All together there were 28 Control dams, 18 Pre dams and 15 P/G dams. Offspring were categorized by sex and group in the studies included male Control (n = 22), Pre (n = 26), P/G (n = 16); Female: Control (n = 47), Pre (n = 22), P/G (n = 14). The individual number of animals per assay is listed in figures. After spontaneous birth the dams were left undisturbed with their offspring for 24 hours. After 24 hours, the P/G group were switched from HFD to ND. Litter sizes were standardized to 6–8 mice each. All offspring were weaned from the mothers at 21 days postpartum and were continued on ND.

Both male and female offspring were assessed in this study. The offspring had weekly weights from birth. At approximately 8 weeks of age, the offspring underwent a 6-hour fasted glucose tolerance test. In addition, a fasted serum insulin level was measured with an ultrasensitive insulin ELISA kit (Crystal Chem, Elk Grove Village, IL). At 13 weeks of age, half of the offspring from each diet group were started on HFD to mimic an obesogenic postnatal diet. At 22 weeks of age (10 weeks of HFD for those on postnatal HFD), all offspring underwent a 6-hour fasted glucose tolerance test. The endpoint analysis was performed at 24 weeks of age (12 weeks of HFD for those on postnatal HFD). Tissues, such as subcutaneous inguinal white adipose tissue (IWAT), gonadal white adipose tissue (GWAT), and liver, were excised and weighed for each animal.

### Intraperitoneal glucose tolerance test (IPGTT)

Glucose tolerance tests were performed on offspring at approximately 8 and 22 weeks of age. Mice were fasted in the morning for 6 hours prior to glucose delivery via intraperitoneal injection at a dose of 0.7 g/kg. Glucose levels were determined shortly before the injection (0 minutes), then 30 minutes, 45 minutes, 60 minutes, and 120 minutes after the injection. Blood was obtained via a tail vein and the glucose was measured with a Freestyle Lite glucometer^30^.

### Immunofluorescence microscopy

Adipose tissue was fixed in 1% paraformaldehyde for 24 hours and then transferred to phosphate buffered saline (PBS) for storage and stored at 4 °C. Tissues were prepared with blocking solution (0.3% Triton, 5% bovine serum albumin in PBS). The tissue was stained with polyclonal anti-caveolin (Cell Signaling Technology) and anti-Mac2 (Cell Signaling Technology). Imaging analysis was performed on an OLYMPUS inverted microscope for epifluorescence and captured with an OLYMPUS DP74 charged-coupled device camera. The software used to record the images was OLYMPUS cell Sans Standard software. ImageJ (National Institute of Health, Bethesda, MD) was used to generate the composite images^31^.

### Hematoxylin and eosin (H&E) and Periodic Acid-Schiff (PAS) staining

Adipose tissue and liver tissue were fixed in 10% formalin for 48 hours and then stored in ethanol at room temperature. Tissues were paraffin embedded, sectioned, and stained with H&E using the University of Michigan’s Comprehensive Cancer Center Histology Core. Slides were photographed using an OLYMPUS DP74 charge-coupled device camera and OLYMPUS microscope. The software used was OLYMPUS cellSans Standard software. PAS staining was done according to the PAS staining kit protocol (Cat. No. 395B-1KT, Sigma-Aldrich). Stained slides were mounted using Permount (Cat. No. 17986-01, Electron Microscopy Sciences) and imaged at 10X.

### Adipocyte sizing

Adipocyte size was determined in fixed GWAT samples stained with H&E. An OLYMPUS cellSans Standard software was used to capture multiple TIFF-grayscale images. Adipocyte size was determined in each image using ImageJ (National Institute of Health) to manually delineate adipocyte membranes and measure cross-sectional area^31^. The adipocyte sizes were averaged for each mouse. Approximately 100 adipocytes were imaged per sample.

### Adipose tissue stromal vascular fraction (SVF) isolation and flow cytometry

Analysis of adipose tissue SVF by flow cytometry was performed to evaluate leukocyte populations^31^. The stains used were CD64 PE, CD45.2 e450, and CD11c-APC-Cy7 for ATMs. Gating was performed for macrophage populations and by CD45 gates to determine ATMs^32,33^. Flow cytometry was performed at the University of Michigan Flow Cytometry Core on the BD FACS Aria using Diva Software and then analyzed with FlowJo Software.

### Hepatic triglyceride content

Liver samples were weighed, frozen in liquid nitrogen, and stored at −80 °C. The frozen liver samples were cut to a weight of 50–100 mg, lysed, and homogenized in methanol-butanol liver lysis buffer using a pellet pestle. The solution was dried overnight with a speed-vac. The lipid was then extracted with the addition of chloroform and dried. The residual lipid was then dissolved in butanol. Triglycerides were assessed and quantified using the Infinity Triglyceride Assay Kit (Sigma)^34,35^ and normalized to mass of the liver tissue initially used for assay.

### Maternal lipolysis during gestation and lactation

A separate cohort of breeding mothers was setup under the same condition as previously described. Virgin female and male wild type C57BL/6J mice were purchased from Jackson Laboratories or obtained from breeding pairs with only control ND exposure. The female mice were similar ages +/−2 weeks. The same diet groups were setup. Breeding cages were established at approximately 16 weeks of age, where one female was assigned to one male. Female mice were weighed and had serum collected at the presence of a vaginal plug (Time: 0), on gestation day 7 (G7), gestation day 14 (G14), postnatal day 7 (PN7), and postnatal day 21 (PN21). At the presence of a vaginal plug, the male mouse was separated from the female mouse. After spontaneous birth, the litters remained with their mother until postnatal day 21. Any female mice that did not give birth were excluded from the data analysis.

To determine the degree of lipolysis, serum FFA and glycerol were measured using the collected serum of the mothers as described above. Blood from female (mother) mice was collected around the mid-afternoon because it is typically the time of lowest food consumption thus allowing for consistent measurement^36^. This method was chosen after discussion with veterinary staff about the stress of frequent fasting for multiple time points during gestation and in the immediate post-natal period. Repetitive bouts of fasting would induce stress and potentially risk the maintenance of pregnancy. Serum non-esterified fatty acid (NEFA) was measured using the HR NEFA series (Wako Diagnostics, Richmond, VA). Serum free glycerol was measured using free glycerol determination reagent (Sigma-Aldrich, St. Louis, MO).

### Statistical analysis

Data are expressed as a mean +/− standard error of the mean (SEM). Statistical significance of differences between controls and diet groups were determined using either a parametric one-way ANOVA followed by post-hoc analysis with unpaired two-tailed Student *t* -test or a non-parametric Kruskal-Wallis test if the n was small and distribution hence could not be determined. Differences were considered significant with a p-value less than 0.05. In all cases, “n” refers to the numbers of mice in each diet group.

## Results

### Maternal body weight increases with preconception HFD and is reduced during gestation due to cessation of HFD

**Figure 3:**
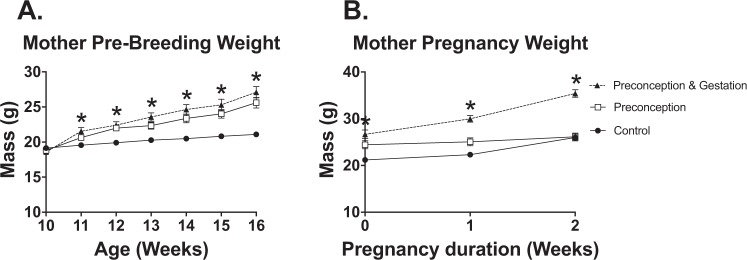
Maternal characteristics prior to pregnancy and during pregnancy. (**A**) Mother pre-breeding weight at ages 10–16 weeks of age, Control (n = 32), Pre (n = 18), P/G (n = 15). (**B**) Pregnancy weight, Control (n = 28), Pre (n = 18), P/G (n = 15). *p value < 0.05. Pre and P/G mothers had expected increase of pre-breeding weight due to HFD. During pregnancy, P/G group continued to gain weight while the Pre group’s weight converged with the Control group.

10-week-old females were started on control or HFD. Over 6 weeks, female mice that were on the HFD had greater weight gain compared to the controls (Fig. 3A). Breeding was started at 16 weeks of age. HFD was discontinued in the Pre group when a vaginal plug was visualized. During the gestation period, the P/G group continued to gain weight above controls while the weight gain subsided in the Pre group. Near the end of the gestation period, the Pre group weight was comparable to the control group (Fig. 3B).

### When maintained on normal diet offspring metabolic phenotypes are similar regardless of maternal diet

**Figure 4:**
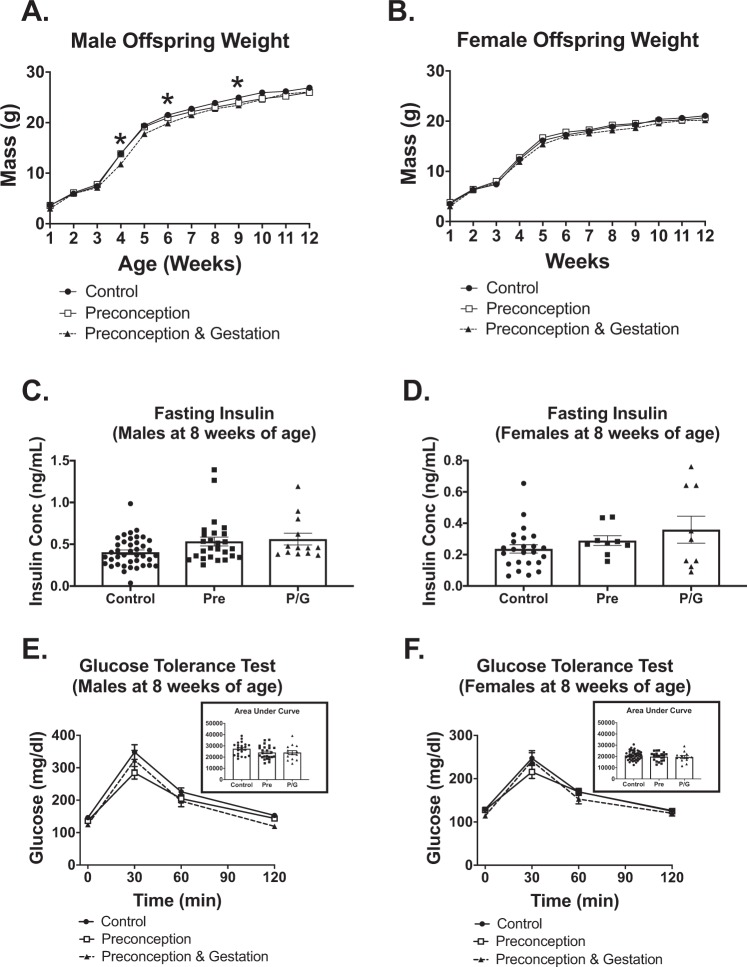
Male and female offspring metabolic characteristics prior to postnatal HFD. Weekly weights from 1–12 weeks of age (**A**) in male and (**B**) in female offspring, Male: Control (n = 22), Pre (n = 26), P/G (n = 16); Female: Control (n = 47), Pre (n = 22), P/G (n = 14). *p < 0.05. Fasting insulin at 8 weeks (**C**) in male and (**D**) in female offspring, Male: Control (n = 40), Pre (n = 26), P/G (n = 12); Female: Control (n = 25), Pre (n = 9), P/G (n = 10). Glucose tolerance test with AUC at 8 weeks (**E**) in male and (**F**) in female offspring, Male: Control (n = 22), Pre (n = 25), P/G (n = 14); Female: Control (n = 43), Pre (n = 22), P/G (n = 12). Among the male groups, P/G intermittently had lower weights. There was no significant difference in fasting insulin and GTT for both male and female.

All offspring were maintained on ND during the first 12 weeks of life. Weekly weights showed that among the males, the P/G group intermittently had significantly lower weight at 4, 6, and 9 weeks of age (Fig. 4A), but overall were of similar weight at 12 weeks of life. There was no significant difference in weight for female offspring (Fig. 4B). In both male and female offspring, metabolic testing at 8 weeks of age showed no significant difference between the fasting insulin levels (Fig. 4C,D) or overall glucose tolerance test as measured by IPGTT and based on area-under-the-curve (AUC) (Fig. 4E,F). These results suggest that a second hit that introduces metabolic stress might be required to induce metabolic changes in the offspring.

### Male offspring from preconception HFD exposure had the greatest weight gain with postnatal HFD

**Figure 5:**
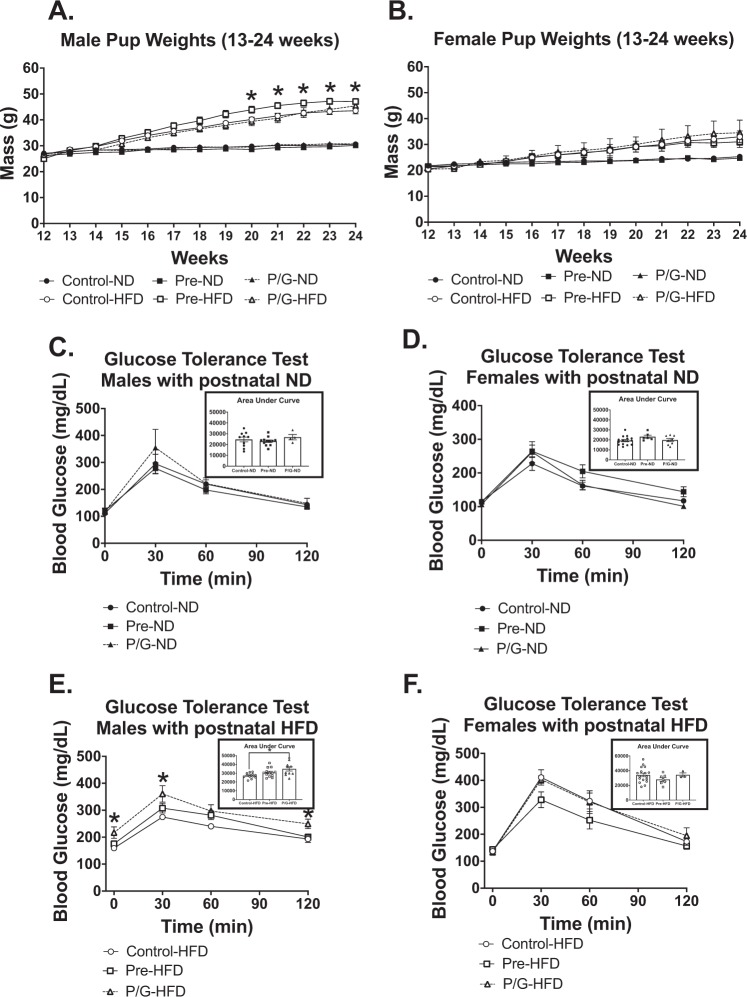
Male and female offspring metabolic characteristics on postnatal ND or postnatal HFD. Weekly weights from 13–24 weeks of age (**A**) in male and (**B**) in female offspring, Male: Control-ND (n = 10), Pre-ND (n = 12), P/G-ND (n = 4), Control-HFD (n = 12), Pre-HFD (n = 14), P/G-HFD (n = 10); Female: Control-ND (n = 6), Pre-ND (n = 6), Control-HFD (n = 17), Pre-HFD (n = 7), P/G-HFD (n = 3). *p < 0.05 GTT and AUC at 22 weeks for groups with postnatal ND (**C**) in male and (**D**) in female offspring, Male: Control-ND (n = 10), Pre-ND (n = 12), P/G-ND (n = 4); Female: Control-ND (n = 15), Pre-ND (n = 6), P/G-ND (n = 9). GTT and AUC at 22 weeks for groups with postnatal HFD (**E**) in male and (**F**) in female offspring. Male: Control-HFD (n = 11), Pre-HFD (n = 14), P/G-HFD (n = 10); Female: Control-ND (n = 17), Pre-ND (n = 7), P/G-ND (n = 3). *p < 0.05. Among the male groups with postnatal HFD, there was a significant increase in body weight, greatest for Pre group from week 20–24. For IPGTT, P/G with postnatal HFD had the worst glucose tolerance.

To determine whether latent programmed phenotypes emerge under situations of a later metabolic stress we challenged the offspring with a HFD exposure. At 12 weeks of age, some of the offspring remained on ND (Control-ND, Pre-ND, and P/G-ND) while other offspring were started on postnatal HFD (Control-HFD, Pre-HFD, and P/G-HFD). Male and female Control-ND, Pre-ND, and P/G-ND did not differ in weight among their sex (Fig. 5A,B). Groups with postnatal HFD gained more weight compared to the groups on postnatal ND. For the males, Pre-HFD mice had the greatest weight gain (statistically significant from week 20–24) (Fig. 5A). For females, there was no significant weight difference among the diet groups with postnatal HFD (Fig. 5B).

### Male offspring from preconception/gestation HFD exposure had worsened glucose tolerance on postnatal HFD

Glucose tolerance testing at 22 weeks was similar among all male and female offspring on postnatal ND (Fig. 5C,D). P/G HFD male offspring had a higher fasting glucose and glucose value at baseline, 30 minutes, and 120 minutes after glucose administration, when compared to Control-HFD. When AUC was compared the P/G HFD group was significantly higher than Control-HFD (Fig. 5E) indicating worsened glucose tolerance. There was no significant difference in glucose tolerance for females with postnatal HFD (Fig. 5F).

### Male offspring from preconception HFD exposure had the greatest body weight, subcutaneous fat pad and liver expansion with postnatal HFD

**Figure 6:**
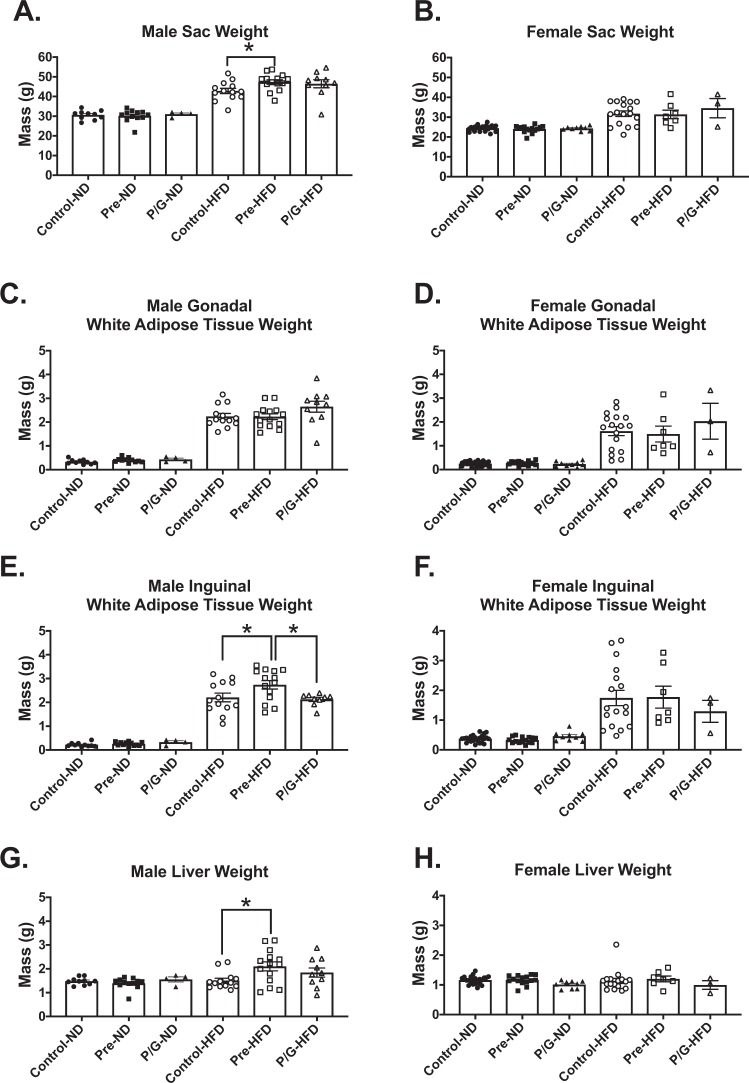
Male and female offspring tissue weights at 24 weeks. Sac weight for the groups with postnatal ND and postnatal HFD (**A**) in male and (**B**) in female offspring. GWAT weight for the groups with postnatal ND and postnatal HFD (**C**) in male and (**D**) in female mice. IWAT weight for the groups with postnatal ND and postnatal HFD (**E**) in male and (**F**) in female. Liver weight for the groups with postnatal ND and postnatal HFD (**G**) in male and (**H**) in female. *p < 0.05. Male: Control-ND (n = 10), Pre-ND (n = 12), P/G-ND (n = 4), Control-HFD (n = 13), Pre-HFD (n = 14), P/G-HFD (n = 10); Female: Control-ND (n = 23), Pre-ND (n = 15), P/G-ND (n = 10), Control-HFD (n = 17), Pre-HFD (n = 7), P/G-HFD (n = 3).

At 24 weeks of age, all offspring were dissected, and body weight, liver, and adipose tissues weights were determined. For males, there was no significant difference among groups with postnatal ND (Fig. 6A,C,E,G). Among the males with postnatal HFD, Pre-HFD mice were the heaviest (Fig. 6A). There was no significant difference in GWAT weight among the different groups (Fig. 6C). Pre-HFD animals had the greatest IWAT (Fig. 6E) and liver weight (Fig. 6G). However, there was no significant difference in body weight or tissue weights for females in the ND or postnatal HFD groups (Fig. 6B,D,F,H).

### Male offspring from preconception/gestation HFD exposure developed more CD11c^−^ macrophages with postnatal HFD

**Figure 7:**
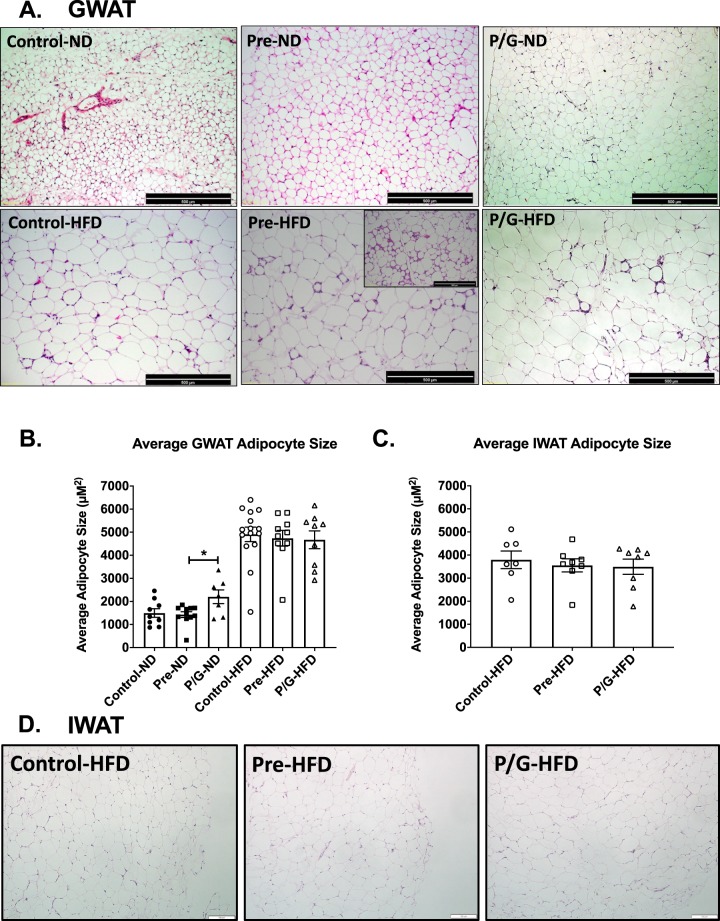
Morphology of GWAT and IWAT in male offspring at 24 weeks of age. (**A**) Hematoxylin and eosin (HE) staining of GWAT of groups (Control, Pre, P/G) with postnatal ND (top row) and with postnatal HFD (bottom row). The Pre-HFD shows different degrees of fibrosis (smaller and larger image). (**B**) Average adipocyte sizes comparing groups with postnatal ND (n = 8 per group). Average adipocyte sizes were all significantly larger with postnatal HFD but there was no significant difference among the postnatal HFD groups, Control-HFD (n = 15), Pre-HFD (n = 10), P/G-HFD (n = 9). (**C**) Average IWAT adipocyte size (n = 8 per group) in the prenatal HFD challenged group. (**D**) H&E staining of IWAT from the prenatal HFD challenged group. *p < 0.05.

Next, we examined the histology of GWAT to investigate adipocyte morphology and inflammation. H&E staining of male GWAT showed that all groups with postnatal HFD have larger adipocytes and findings suggestive of more tissue fibrosis and macrophages compared to groups maintained on ND (Fig. 7A). While there was an increase in the P/G ND GWAT adipocytes, there was no significant difference in average adipocyte size among the Control, Pre, or P/G HFD groups (Fig. 7B). IWAT adipocytes were not different amongst groups as seen by sizing and H&E images (Fig. 7C,D).

**Figure 8:**
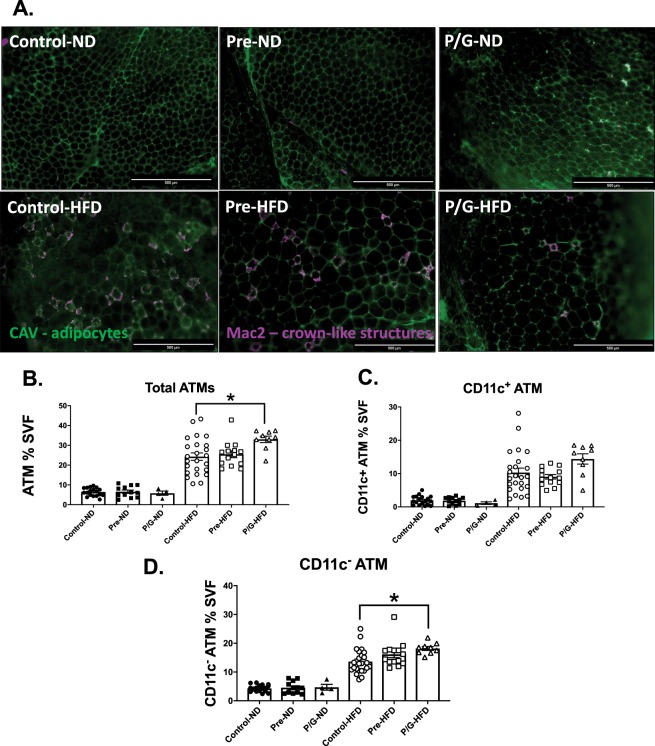
Immunofluorescence and flow cytometry studies of GWAT in male offspring at 24 weeks of age. (**A**) Staining of adipocytes (Caveolin, green) and cluster of macrophage crown-like structures (Mac2, magenta). Groups with postnatal HFD have larger adipocytes and more macrophage crown-like structures. GWAT shows increased crown-like structures with postnatal HFD. The Pre-HFD shows different degrees of crown-like structures (smaller and larger image) (**B**) GWAT ATM of SVF: Control-ND (n = 18), Pre-ND (n = 12), P/G-ND (n = 4), Control-HFD (n = 24), Pre-HFD (n = 14), P/G-HFD (n = 9) (**C**) CD11c^+^ ATMs of SVF: Control-ND (n = 18), Pre-ND (n = 12), P/G-ND (n = 4), Control-HFD (n = 24), Pre-HFD (n = 14), P/G-HFD (n = 9) (**D**) CD11c^−^ ATM of SVF: Control-ND (n = 18), Pre-ND (n = 12), P/G-ND (n = 4), Control-HFD (n = 24), Pre-HFD (n = 14), P/G-HFD (n = 9) *p < 0.05. Flow cytometry of male GWAT at 24 weeks of age show an increase in CD11c^−^ ATMs in P/G group.

Immunofluorescence imaging of ATMs in male GWAT on postnatal HFD showed increased clusters of macrophage crown-like structures (CLS), an evidence of recruited macrophages (Fig. 8A). Flow cytometry of male GWAT was performed to quantify and characterize the ATMs. Our analyses showed that groups with postnatal ND had no significant difference for total ATMs, CD11c^+^ ATMs, and CD11c^−^ ATMs (Fig. 8B–D). Among the postnatal HFD groups, the P/G group had the greatest total ATMs, specifically CD11c^−^ ATMs (Fig. 8B,D). However, there was no significant difference in CD11c^+^ ATMs (Fig. 8C).

**Figure 9:**
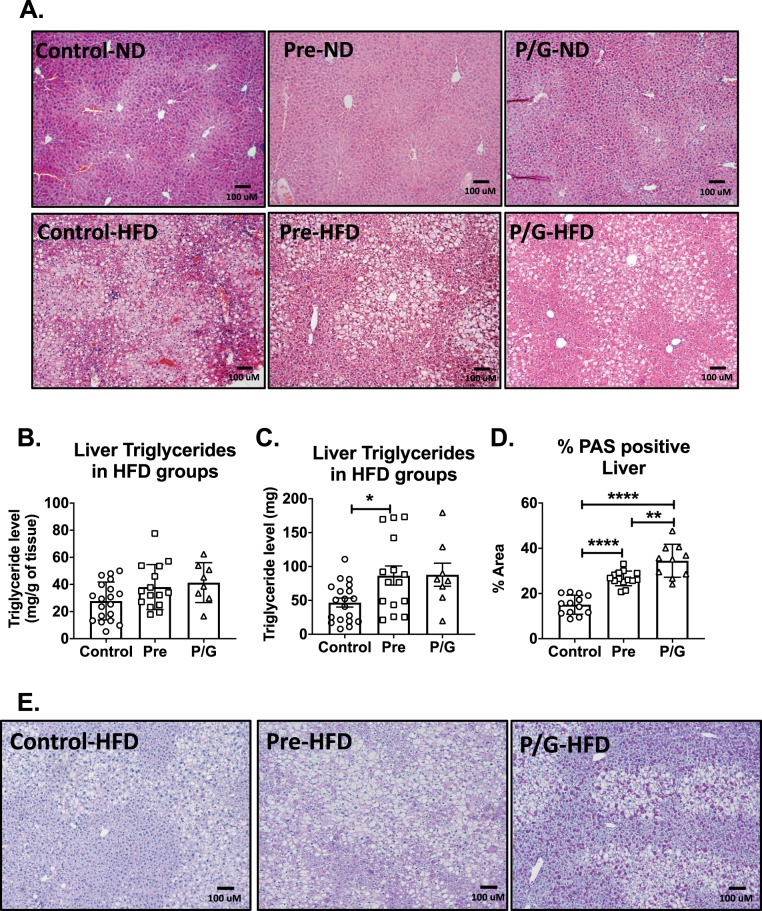
Liver morphology and triglyceride content in male offspring. (**A**) Hematoxylin and eosin (HE) staining of liver of male groups (Control, Pre, P/G) with postnatal ND (top row) and with postnatal HFD (bottom row). There are increased lipid globules with postnatal diet. (**B**) Comparison of liver triglyceride content adjusted for liver weight (milligram of triglyceride per gram of liver) between the male groups with postnatal HFD. Control-HFD (n = 18), Pre-HFD (n = 15), P/G-HFD (n = 8). (**C**) Comparison of absolute liver triglyceride content (measured in milligram) between the male groups with postnatal HFD. Control-HFD (n = 18), Pre-HFD (n = 15), P/G-HFD (n = 8). There is no significant difference in liver triglyceride content among groups with postnatal HFD. (**D**) Quantification of % Area of liver stained with PAS from postnatal HFD groups and (**E**) representative images PAS staining of liver of postnatal HFD groups.

Given differences in adiposity and liver mass, we next evaluated hepatic steatosis. There was increased steatosis in the postnatal HFD groups as demonstrated in H&E images (Fig. 9A). When liver triglyceride content (milligram per gram of liver tissue) was quantified, there was no significant difference between the groups exposed to postnatal HFD (Fig. 9B). When normalized to total liver mass, the liver triglyceride content (milligram) was significantly higher in triglyceride in the Pre-HFD group compared to control (Fig. 9C). Periodic acid-Schiff (PAS) staining was performed to determine if the areas of clearing seen in the post-natal HFD groups with H&E may be due to glycogen deposition. Quantitation of the PAS staining demonstrated a larger quantified area with PAS staining in the Pre HFD compared to control while the P/G HFD group had the largest area of PAS staining (Fig. 9D). Staining demonstrated much brighter appearing material within the liver of animals from the P/G HFD group (Fig. 9E). Overall, these results demonstrated that the Pre-HFD group had expanded liver triglyceride storage contributing to liver mass while the P/G HFD group had a differential response in the liver leading to enhanced PAS staining.

### Mothers with preconception HFD exposure have higher GWAT expansion but lower serum glycerol compared to mothers continued on HFD during gestation

We next investigated if maternal lipolysis varied based on the timing of the diet switch. Female (mother) weights and serum non-esterified fatty acid (NEFA) and free glycerol were measured at the start of breeding (0), early gestation (gestation day 7 [G7]), late gestation (gestation day 14 [G14]), lactation day 7 (PN7), and lactation day 21 (PN21).

**Figure 10:**
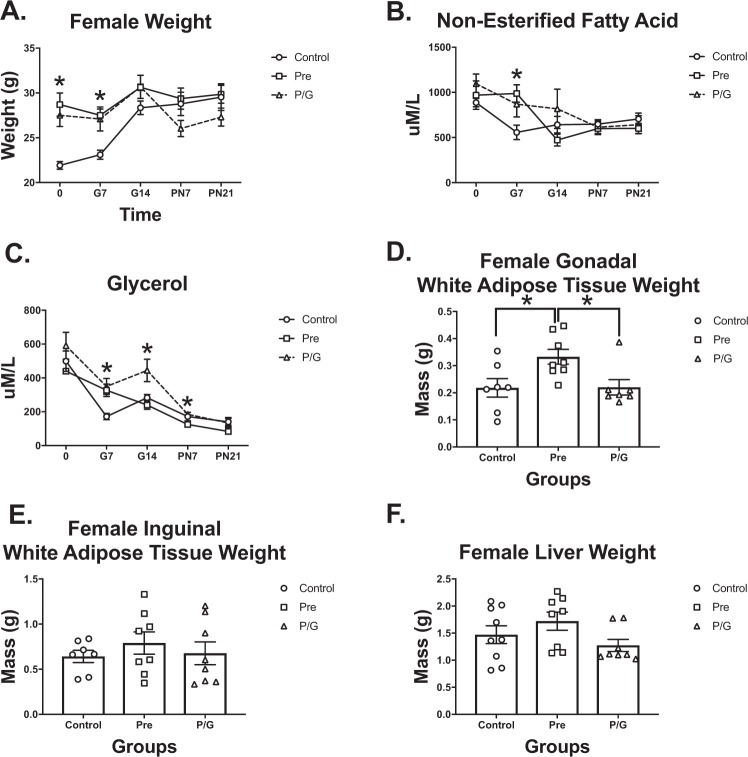
Maternal characteristics at the start of pregnancy (0) to postnatal day 21 (PN21) Following 6 weeks of HFD breeding cages were established and (**A**) mother weight from 0 thru PN21 charted for all groups. Control (n = 7), Pre (n = 8), P/G (n = 8). (**B**) Non-esterified fatty acid from 0 thru PN21. Control (n = 7), Pre (n = 8), P/G (n = 8) (**C**) Glycerol from 0 thru PN21. Control (n = 7), Pre (n = 8), P/G (n = 8) (**D**) IWAT weight. Control (n = 7), Pre (n = 8), P/G (n = 8) (**E**) GWAT weight. Control (n = 7), Pre (n = 8), P/G (n = 8) (**F**) Liver weight. Control (n = 7), Pre (n = 8), P/G (n = 8) *p < 0.05. During pregnancy, Pre and P/G group continued to gain weight. P/G lost weight when HFD was discontinued at time of birth. There was a significant difference in NEFA at G7, in glycerol from G7-P7. The pre-pregnancy HFD mothers (Pre) mother had the greatest GWAT weight at the end of the lactation period.

The Pre and P/G groups were significantly heavier at 0 and G7 when compared to Control. Although HFD was discontinued for the Pre group at the start of breeding, this group had a similar weight pattern like the P/G group during gestation. When HFD was discontinued at birth for P/G, there was a decline in weight (Fig. 10A). NEFA was significantly higher in Pre when compared to Control at G7 (Fig. 10B). For glycerol, P/G was greater than Control at G7 and G14, and greater than Pre at G14 and P7 (Fig. 10C). At the end of the lactation period (PN21), the mothers were dissected. There was no significant difference in body weight, IWAT or liver mass (Fig. 10E,F), but the Pre had heavier GWAT (Fig. 10D). Overall, this data suggests that offspring from the P/G mothers may be exposed to more glycerol during gestation and lactation, while offspring from the Pre mothers are exposed to more NEFAs during the early gestation window which may also lead to an accumulation of visceral adiposity for mothers.

## Discussion

In this study, we evaluated the developmental programming effects of maternal obesity during critical windows of development on offspring metabolism and adipose tissue health. Our results showed that the prenatal and pregnancy window can have independent programming impacts on offspring adiposity and metabolic inflammation. Maternal obesity during the preconception period only led to male offspring with greater weight gain and larger inguinal (subcutaneous) fat depot when a postnatal HFD was introduced. On the other hand, maternal obesity that started prior to breeding and maintained through pregnancy resulted in male offspring with greater glucose intolerance and increased number of ATMs in the gonadal (visceral) fat depot when a postnatal HFD was introduced.

It is believed that within the developmental programming field, the fetus adapts to an adverse intrauterine environment to improve its immediate chances for survival. The adaptive change in fetal physiology may be beneficial for short-term survival *in utero*, but becomes maladaptive later in life^37^. Consistent with this, our study shows that when a “second-hit” stressor (obesogenic diet) was introduced, the latent programming effects from maternal obesity were revealed. We observed that the preconception developmental window has the greatest impact on offspring fat distribution. The male offspring from this group had the largest IWAT (subcutaneous) fat. This seems appropriate because subcutaneous fat functions as a normal physiological buffer for excess energy intake and is a site where excess free fatty acids and glycerol are stored as triglycerides^38^. However, when lipid supply exceeds the storage capacity of the subcutaneous fat, then fat accumulates in areas outside the subcutaneous tissue such as visceral tissue (a fat depot that is more prone to metabolic inflammation) and liver^39^. Our result could suggest that HFD exposure only during the preconception window reprogrammed the offspring to have an expanded storage capacity in their subcutaneous adipose tissue instead of the visceral adipose tissue. This might delay the negative metabolic effects, but this group also had greater liver mass and total liver triglycerides with postnatal HFD. Prior studies have demonstrated that *in utero* and lactation HFD exposure increased hepatocyte proliferation and hepatosteatosis in offspring^40^, but further studies are needed to determine if the pre-pregnancy window has a greater influence on changing liver health. In addition, PAS staining demonstrated brighter staining in the P/G HFD offspring suggesting that this group that was exposed to HFD and during early development had a secondary response to store more liver glycogen. This response may be a protective one, given that liver glycogen has been demonstrated to decrease food intake, regulate glucose homeostasis, and improve inflammation^41,42^. This potentially protective response in the face of continued HFD during the pregnancy window requires further investigation.

In this study we also observed that postnatal HFD led to worsened glucose tolerance and increased adipose inflammation in male offspring born from mothers with preconception and gestation HFD exposure. This is consistent with the notion that macrophages within the adipose tissue generate a sustained proinflammatory tone which negatively affect adipocyte insulin sensitivity^43^. Traditionally chronic obesity induced inflammation has been associated with increased recruitment of ATMs that produce proinflammatory cytokines and activate other leukocyte populations. These recruited ATMs prominently express a CD11c^+^ marker and form CLSs around dead or dying adipocytes. ATMs that express a CD11c^−^ marker are generally resident ATMs thought to have deposited during embryonic development^27,44^. While we anticipated that postnatal HFD challenge would increase the presence of ATMs in the male offspring, we expected that it would be due to more recruitment of CD11c^+^ ATMs in the visceral fat depot. However, instead there was an increase in resident CD11c^−^ ATMs. Potentially the prolonged exposure to the obesogenic diet during the gestation period influenced the yolk sac derived leukocytes^9,45^, leading to more resident macrophages that were able to expand when these animals were re-challenged to a HFD. A limitation of this study was that the ATMs were not quantified and characterized in the IWAT (subcutaneous) fat depot. This would be helpful to determine whether resident ATMs in the P/G HFD group would also similarly respond to what was seen in GWAT. However, H&E staining of the IWAT did not demonstrate enhanced CLSs suggesting that CD11c^+^ ATMs are likely not different amongst groups. Another limitation of our study is that we did not explore other ramifications of this tissue inflammatory tone such as effects on the dendritic cell and T cell populations which may also have been programmed during exposure in these windows of immune system development.

Another finding in our studies is that male offspring were the only animals influenced by the maternal obesity. Our results are consistent with prior studies that showed a sex difference in developmental programming, where the male offspring experienced a greater effect from the insult compared to females^7^. Our prior work demonstrates that there is generally a sexual dimorphism in adiposity, metabolic, and inflammatory responses to high fat diet^46^. Specifically, the ATM phenotype in females on HFD are predominantly the CD11c^−^ ATMs type. Females have enhanced adipogenesis, adipocyte hypertrophy and regulatory inflammatory responses to a HFD even with lipolysis induction^47^. The result of this is improved insulin sensitivity in obese females. We hypothesize that a similar protective effect might be present in females *in utero* and hence female offspring are more protected from these programmed effects. A limitation to our studies is that after the “second hit” of HFD females were relatively overall resistant which may also be due to the strain specific effects of using C57Bl6 mice where females are more resistant to HFD^48^. Even with these caveats, this knowledge is critical because it provides an important risk factor which may be an incentive for earlier intervention if the expecting mother has knowledge of the sex of the offspring. Further studies are needed to understand the mechanisms that lead to these sex differences in programming.

To identify a possible mechanism for the programming effects, we turned our focus to changes in the mothers’ metabolic profile. There are dramatic changes in metabolism during pregnancy that not only influence the mother but also the placenta and its transfer of nutrients. Placental influences have previously been reported to be sexually dimorphic and may explain why a male fetus is affected while the female fetus appears protected^49,50^. There is naturally an increase in fat accumulation, insulin production, insulin resistance, and circulating lipid levels to promote offspring growth^51^. This helps the mother to produce enough nutrition for herself and for her fetus. Therefore, we speculated that there might be a difference in the degree of lipolysis during pregnancy and lactation that affects the circulating FFA and glycerol level being transferred to the offspring. During lactation, there are further changes in lipid metabolism to allow for mobilization of lipids for milk synthesis^52^. In our study, we observed a difference in NEFA and glycerol between the Pre and P/G mothers during the mid to late gestation period. Despite not having a significant difference in weight, there was greater NEFA and glycerol levels when HFD was maintained through pregnancy. This suggests that the fetus received a variable amount of lipids from their mothers depending on the diet, leading to different adipose and liver fat accumulation and metabolic inflammation in the offspring. It is important to note that the mid to late gestation period is a time when adipocytes are forming and when the liver acts as a source for hematopoiesis.

Another limitation of our study is that we did not evaluate the serum fatty acid and glycerol level in the fetus. This would have been useful as a surrogate marker of placental transfer of fatty acids from the mother to the fetus. In future work, direct assessment of the placenta in these groups is necessary to understand the contribution of placental pathology on offspring programming. This is especially important as the placenta may be a major source of inflammatory response with lipid transfer and also a major source for establishing the type of inflammatory response in offspring^53–55^.

## Conclusion

Our study demonstrates that maternal obesity prior to conception has a programming effect on offspring weight, subcutaneous fat pad expansion and liver weight. It also demonstrates and that maternal obesity prior to conception and continued through gestation increases offspring risk for adipose tissue inflammation and metabolic dysfunction. Overall, this study demonstrates that prenatal and pregnancy windows have independent effects on programming offspring body composition and immune cell phenotypic states. A potential mechanism for this is the degree of lipolysis occurring during the gestation period, affecting the nutritional exposure of the fetus to lipids, but further studies will be needed to understand the factors from maternal lipolysis to that regulate tissue programming.

With the rapid rise of obesity in mothers and their children, and the concern of its trans-generational effect, it is important to better understand the critical windows of development and their independent influences on the risk for metabolic disease. By having a better insight into which developmental windows are most affected by maternal obesity, we can generate future studies that are even more impactful. Translation of this knowledge to a clinical application of how to approach anticipatory guidance with prenatal care in women of reproductive age might not only improve a mother’s health but also the health of her children.

## Data Availability

Datasets generated during and/or analyzed during the current study are not publicly available but are available from the corresponding author on reasonable request.
